# BCG Vaccination Prevents Reactivation of Latent Lymphatic Murine Tuberculosis Independently of CD4^+^ T Cells

**DOI:** 10.3389/fimmu.2019.00532

**Published:** 2019-03-21

**Authors:** Harindra D. Sathkumara, Saparna Pai, Michel de Jesús Aceves-Sánchez, Natkunam Ketheesan, Mario Alberto Flores-Valdez, Andreas Kupz

**Affiliations:** ^1^Centre for Molecular Therapeutics, Australian Institute of Tropical Health and Medicine, James Cook University, Cairns & Townsville, QLD, Australia; ^2^Centro de Investigación y Asistencia en Tecnología y diseño del Estado de Jalisco, A.C., Biotecnología Médica y Farmacéutica, Guadalajara, Mexico; ^3^Science and Technology, University of New England, Armidale, NSW, Australia

**Keywords:** tuberculosis, BCG, vaccines, CD4 T cells, reactivation, BCGΔBCG1419c, latent tuberculosis

## Abstract

Tuberculosis (TB) is a major global public health problem causing significant mortality and morbidity. In addition to ~10.4 million cases of active TB annually, it is estimated that about two billion people are latently infected with *Mycobacterium tuberculosis* (*Mtb*), the causative agent of TB. Reactivation of latent *Mtb* infection is the leading cause of death in patients with immunodeficiency virus (HIV) infection. The low efficiency of the only licensed anti-TB vaccine, Bacille Calmette–Guérin (BCG) to reduce pulmonary TB in adults contributes to this problem. Here we investigated if vaccination with conventional BCG or the genetically modified experimental BCGΔBCG1419c strain can prevent reactivation of latent lymphatic TB in a mouse model of induced reactivation, following the depletion of CD4^+^ T cells, as it occurs in HIV^+^ individuals. Vaccination with conventional BCG or BCGΔBCG1419c prevented reactivation of *Mtb* from the infected lymph node and the systemic spread of *Mtb* to spleen and lung. Prevention of reactivation was independent of vaccination route and was accompanied by reduced levels of circulating inflammatory cytokines and the absence of lung pathology. Our results demonstrate that vaccine-induced CD4^+^ T cells are not essential to prevent reactivation of latent lymphatic murine TB, and highlight the need to better understand how non-CD4^+^ immune cell populations participate in protective immune responses to control latent TB.

## Introduction

Tuberculosis (TB) affects ~10.4 million people annually and is associated with 1.7 million deaths per year (1). Despite the availability of effective anti-TB drugs, poor adherence to long treatment regimens contributes to the emergence of multi drug-resistant strains of *Mycobacterium tuberculosis* (*Mtb*), the causative agent of TB (1). Additionally, it is estimated that about 2 billion people are latently infected with *Mtb* without showing signs of active disease (1).

The immune system usually contains *Mtb* infection through the formation of granulomatous lesions (2). However, immunosuppressed individuals such as those who have co-morbid human immunodeficiency virus (HIV) infection or diabetes mellitus have an impaired ability to control latent TB infection (LTBI) (3), resulting in active disease and transmission. In fact, reactivation of LTBI is the number one cause of death in HIV co-infected individuals (4, 5). The gradual decline of CD4^+^ T cells, the hallmark of HIV infection, is believed to be a major contributing factor in LTBI reactivation (2). CD4^+^ T cells are a major source of interferon gamma (IFN-γ), a critical cytokine for TB control, and essential for the structural integrity of the granulomas (6). However, the precise role CD4^+^ T cells play in immunity to TB remains a matter of debate (7, 8). In this context, it is interesting that although anti-retroviral therapy largely restores CD4^+^ T cell numbers, the increased risk for reactivation of LTBI is only partially diminished (9), and reactivation of LTBI often occurs early after HIV infection (10). Furthermore, in a macaque model of TB/SIV co-infection, suppression of LTBI reactivation was shown to be independent of CD4^+^ T cells in at least one third of animals (11). In addition, it was very recently demonstrated that a higher monocyte and macrophage turnover was responsible for LTBI reactivation in macaques co-infected with *Mtb* and simian immunodeficiency virus (SIV) (12). Collectively, these findings further challenge the assumption that CD4^+^ T cells are irreplaceable in TB.

The only licensed TB vaccine, Bacille Calmette–Guérin (BCG) is universally used. BCG efficiently prevents miliary and meningeal TB in children, but shows low efficacy against pulmonary TB in adults (13), and hence does not prevent the transmission cycle (14). Over the last decades several new TB vaccines have been developed with a few currently undergoing clinical trials (15). The important role of IFN-γ-secreting CD4^+^ T cells in animal studies, has led to cognate activation and expansion of *Mtb*-specific CD4^+^ T cells through the use of immunodominant *Mtb* antigens being the main strategy for many new TB vaccines under development (16). However, the recent failures of the TB vaccine candidate MVA85A, (17, 18) highlight the need to rethink TB vaccine design and to identify CD4-independent mechanisms that contribute to control of TB. Importantly, it has become increasingly clear that the immunological correlates of vaccine induced protection against *Mtb* are not fully understood and seem to differ between experimental TB vaccines (7, 8). BCG is administered intradermally in early childhood and most TB vaccine candidates in clinical trials are also administered intradermally (19, 20). However, a shortcoming of intradermal BCG administration is the development of weak memory lymphocyte responses, which lack mucosal homing chemokine receptors, such as CCR5 and CXCR3, that allow migration to the lung, the initial site of *Mtb* infection (21). To match the route of vaccination to the route of natural infection, mucosal vaccination into the lung has attracted renewed interest (9, 22–24). It is now clear that vaccination directly into the respiratory tract (aerosol, i.n. and i.t.) generates more protective lung-residing memory T cells (9, 22, 25).

Recombinant BCG strains and attenuated *Mtb* strains have received significant attention as potential replacement vaccines for BCG (13, 26). Live vaccines often elicit a broader immune response compared to protein-based formulations and do not require an adjuvant. The recombinant BCG Δ*ureC::hly* (VPM1002), and the attenuated *Mtb* Δ*phoP* Δ*fadD26* (MTBVAC) are currently undergoing testing in various clinical trial stages (27, 28). Other experimental live recombinant BCG vaccines, such as BCGΔBCG1419c, BCG Δ*zmp1*, and BCG::ESX-1^Mar^, have shown promising results in animal models but have not yet reached human trials (29–32). Similarly, attenuated *Mtb* strains, such as *Mtb* Δ*sigH* (33) and *Mtb* ΔRD1 Δ*panCD* (34) showed increased protection, improved safety and better antigen-specific immune responses in various animal models. The live attenuated BCG-based vaccine candidate BCGΔBCG1419c was developed following the hypothesis that chronic mycobacterial infections and LTBI reproduce aspects of biofilm-formation, and contain different antigens compared to planktonic bacteria (35, 36). BCGΔBCG1419c showed improved protection against chronic *Mtb* infection in BALB/c mice and prevented reactivation from latent-like infection of B6D2F1 mice (29), as well as improved protection against chronic *Mtb* infection of C57BL/6 mice, compared to parental BCG (37). Recently, proteomic comparison of BCGΔBCG1419c has shown that in comparison to BCG, it slightly reduces its production of antigenic proteins such as PstS2, HbhA, CFP17, DnaK, and 35 KDa antigen (38) and transcriptomic comparison of the same strains showed that BCGΔBCG1419c had reduced expression of *groEL1, kasA, fas, fabD, acpM*, and *kasB*, involved in mycolic acid biosynthesis, as well as reduced transcription of genes *hspX, groEL2*, and *groES*, which encode for antigenic chaperones (37), globally contributing to a reduced inflammatory environment during chronic infection.

Several meta-analyses of human studies have found that BCG vaccination protects against active tuberculosis (39–45). However, due to a lack of long-term human follow-up studies and the lack of studies regarding LTBI reactivation in the animal model that most closely resembles LTBI, the non-human primate, in the context of vaccination solely with BCG, it is currently unknown if BCG vaccination also impacts on the reactivation dynamics of LTBI in the context of HIV. Recent mathematical modeling data provided new evidence on the global prevalence of LTBI (46), but fell short of estimating the role of BCG vaccination on LTBI reactivation. A very recent study also found no difference in the prevalence of LTBI in the UK, where the relative incidence between BCG vaccinated and naïve people was compared (47). Another very recent report predicted the possible estimation of the role of BCG vaccination on reactivation from LTBI, but the final outcome is yet to be reported (48). In a statistically underpowered Taiwanese study the percentage of T-SPOT.TB positive, HIV-infected patients, was almost 50% less in people showing a BCG scar compared with those with no evidence of BCG vaccination, therefore suggesting BCG reduces reactivation from LTBI in a HIV setting (49). Hence, in order to better understand the correlates of BCG-induced protection against LTBI reactivation, better models and a deeper understanding of the underlying immune response are required.

Non-human primate models for *Mtb*/SIV co-infection closely resemble human physiology, but are associated with substantial ethical, financial, and logistical limitations (50). We have recently described a new tractable mouse model to study reactivation of LTBI, following the loss of CD4^+^ T cells similar to what occurs in HIV co-infection in humans (51). In this model intradermally (i.d.) infected C57BL/6 mice contain *Mtb* within the local draining lymph nodes (LN) until depletion of CD4^+^ cells occurs, thereby mimicking the reactivation of LTBI following HIV infection. In contrast, *Mtb*-infected CD4-deficient mice and MHC-II deficient mice do not recapitulate the latent aspect of LTBI but rather only show exacerbated disease relative to C57BL/6 mice (52). It was recently proposed that TB has characteristic features of lymphatic diseases and that pulmonary pathology may primarily serve disease transmission (53). The hypothesis that *Mtb* persistence may occur in the lymphatics is supported by historical descriptions of TB cases, experimental models and observations of TB in non-human hosts (53). This model is also supported by recent findings that *Mtb* persists in bone marrow stem cells (54–57). Additional evidence for the importance of lymphatic persistence in LTBI has recently also been provided in high-profile NHP studies (11, 58). In this context, the robust mouse LTBI reactivation model presents a much needed alternative to study LTBI compared to logistically challenging NHP models, and provides an opportunity to thoroughly investigate the importance of CD4^+^ T cell-independent strategies for TB vaccination, which are likely to significantly contribute to the immune response elicited by a broadly protective vaccine.

Using this model, the present study examined (i) if reactivation of murine lymphatic LTBI following the loss of CD4^+^ T cells can be prevented by prior BCG vaccination, (ii) if reactivation dynamics differ between vaccination routes, and (iii) if the genetically modified BCGΔBCG1419c strain can reduce reactivation from lymphatic LTBI in this model.

## Materials and Methods

### Mice

Female C57BL/6 mice were bred and maintained in the animal facilities of the Australian Institute of Tropical Health and Medicine at James Cook University, Australia. Mice were 6–8 weeks old at the time of vaccination, and maintained in a biosafety level 3 facility under specific pathogen free conditions.

### Bacteria

BCG Pasteur, BCGΔBCG1419c and *Mtb* H37Rv were grown in Middlebrook 7H9 broth (BD Biosciences) supplemented with 0.2% glycerol, 0.05% Tween 80, and 10% ADC enrichment (BD Biosciences). Mid-logarithmic cultures were harvested, washed in PBS and stored at −80°C.

### Vaccinations and Infections

C57BL/6 mice were immunized with 5 × 10^5^ (i.t.) or 1 × 10^6^ (s.c.) CFUs as described previously (25). For i.t. vaccination mice were anesthetized via i.p. injections of Xylazine (5 mg/kg) and Ketamine (50 mg/kg). Subsequently, the tongue was pulled out sideways, the inoculum was administered into the oral cavity and the nostrils were covered to direct the inoculum into the trachea. 60 days after vaccination, mice were anesthetized via i.p. injections of Xylazine (5 mg/kg) and Ketamine (50 mg/kg) and were subsequently infected i.d. with 1 × 10^2^ CFU *Mtb* H37Rv in the ear dermis in a volume of 50 μl.

### Enumeration of Bacteria

At designated time points, lungs, spleen, and LNs were aseptically removed. One third of the lung, half of the spleen and two LNs were homogenized in sampling bags (Nasco Whirl-Pak®) containing 1 ml of PBS supplemented with 0.05% Tween 80. Serial dilutions of tissue homogenates were plated onto Middlebrook 7H11 agar supplemented with 10% OADC Enrichment (BD Biosciences) and ampicillin (25 μg/ml) or hygromycin (50 μg/ml). CFU were determined after 3–4 weeks incubation at 37°C based on dilution factor and organ size.

### Antibody-Mediated Depletion of CD4^+^ T Cells

CD4^+^ T cells were depleted from C57BL/6 mice by weekly i.p. injections of 200 μg monoclonal antibody (mAb) against CD4 (clone GK1.5, BioXCell, NH). The first injection was given immediately after *Mtb* infection.

### Cell Isolation

Intra-airway luminal cells were removed from the lung by bronchial lavage. Spleen and lymph nodes were aseptically removed. Lungs were perfused with PBS, mechanically disrupted and digested for 30 min with RPMI 1640 medium supplemented with glutamine, Na-pyruvate, 2-ME, penicillin, streptomycin, 10% heat-inactivated FCS, collagenase D (Roche) and collagenase type VIII (Sigma-Aldrich). Single cell suspensions were prepared by passing organs through a 70 μm cell strainer and red blood cell lysis.

### Flow Cytometry

Viable, red blood cell-depleted single splenocytes were stained with mAb (all from BD Pharmingen) against CD4 (RM4-5), CD8α (53-6.7), CD3 (500A2), CD44 (1M7) and NKp46 (29A1.4), CD69 (H1.2F3), CD103 (M290), CD62L (MEL-14). After washing the cells, samples were analyzed using a FortessaX20 analyzer (BD Biosciences, CA). Fixable Viability stain 780 (BD) was used to exclude dead cells. Cell numbers were enumerated by spiking single cell solutions with blank calibration particles and calculated based on volume and organ size as described previously (59).

### Histology

The left lung lobes were collected aseptically, fixed overnight with 4% w/v paraformaldehyde and embedded in paraffin. Two-micrometers sections were stained with H&E and cell infiltration was enumerated using ImageJ (60). The total surface area of the left lung lobe was measured, followed by measuring all areas of dense cell infiltration within the same lobe. Subsequently, the proportion of cell infiltration relative to total surface area was calculated.

### Multiplex Determination of Cytokines

Blood for serum analysis was collected in serum separator tubes (BD), left for 30 min at room temperature and spun at 12,000 g for 3 min. Sera were stored at −20°C until analysis. Measurements were performed using a multiplex immunoassay kit (MagPix). Heat maps for cytokine expression were prepared with Microsoft Excel by plotting the fold increase of the mean cytokine level relative to unvaccinated and untreated animals (**Figures 2D**, **3D**).

### Data and Statistical Analysis

Flow cytometry data was analyzed using FlowJo software (Treestar, CA). Statistical analysis was performed using GraphPad Prism Version 7, GraphPad software, San Diego, CA as indicated in individual figure legends. One-way analysis of variance (ANOVA) followed by the Dunnett's or Tukey's multiple comparison test was used. A *P* < 0.05 was considered significant.

## Results

### Vaccination Profiling

**Figure 1:**
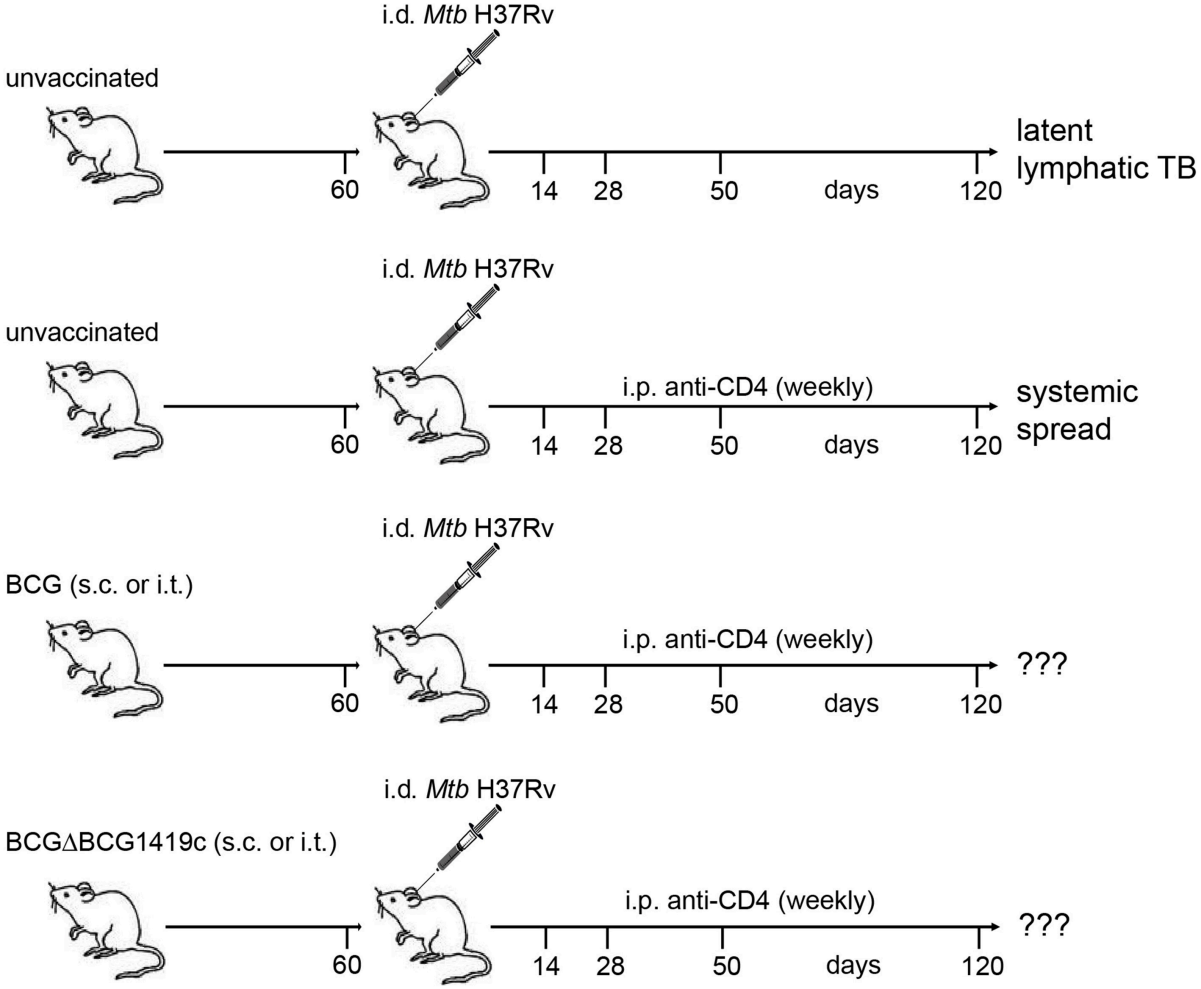
Schematic representation of infection model. Naïve C57BL/6 mice were vaccinated with BCG or BCGΔBCG1419c via the s.c. or i.t. route. Sixty days after vaccination a group of mice was sacrificed and assessed for clearance of the vaccine strain, cellular immune responses, lung histology, and serum cytokines/chemokines. The remaining mice were infected i.d. with *Mtb* H37Rv in the ear dermis. At weekly intervals, mice received i.p. injections of a mAb against mouse CD4 (GK1.5) or PBS. On days 14, 28, 50, and 120 p.i mice were sacrificed. Ear-draining LNs, spleen and lung were assessed for numbers of viable *Mtb*, T cell populations by FACS, lung histology and serum cytokines. Unvaccinated mice without anti-CD4 treatment served as control for latent lymphatic TB and unvaccinated mice treated with anti-CD4 were used as control for reactivation of latent lymphatic infection and systemic spread. BCG, Bacille Calmette–Guérin; *Mtb, Mycobacterium tuberculosis*; TB, tuberculosis; s.c., subcutaneous; i.t., intratracheal; i.p., intraperitoneal; i.d., intradermal.

To determine if BCG vaccination can prevent reactivation of lymphatic murine LTBI following the loss of CD4^+^ cells, we vaccinated C57BL/6 mice with two different strains of BCG: BCG Pasteur (hereafter referred to as BCG) and a BCG Pasteur strain deficient in the gene *BCG1419c* (hereafter referred to as BCGΔBCG1419c). BCGΔBCG1419c vaccination reduced lung pathology and *Mtb* replication in 3 mouse models (29, 35, 37), two of which resemble chronic infection, and the other one resembling reactivation from latent infection. Here, we further evaluated BCGΔBCG1419c's potential as a vaccine candidate against infection in a model resembling reactivation of LTBI upon CD4 deprivation. Mice received the vaccines either as a parenteral subcutaneous (s.c.) injection into the tail base or directly into the lung via intratracheal (i.t.) vaccination (Figure 1).

**Figure 2:**
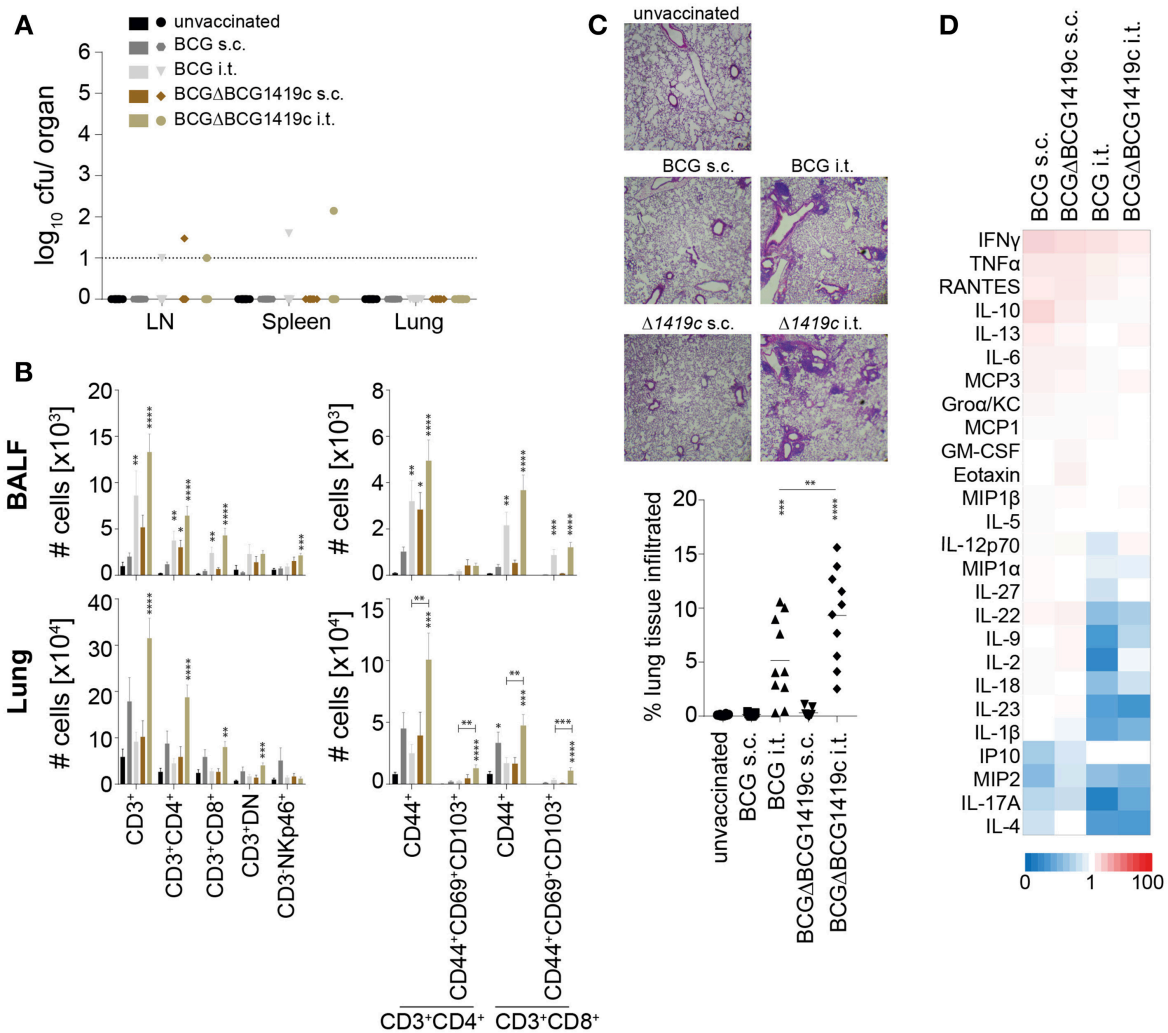
Immune profiling prior to *Mtb* infection. Sixty days after vaccination with BCG or BCGΔBCG1419c mice were assessed for: **(A)** Number of viable BCG in inguinal LN, spleen and lung; **(B)** Number of total CD3^+^, CD3^+^CD4^+^, CD3^+^CD8^+^, CD3^+^CD4^−^CD8^−^ (DN) and CD3^−^NKp46^+^ cells (left plots), as well as numbers of total CD44^+^, CD44^+^CD69^+^CD103^+^ cells amongst CD3^+^CD4^+^ and CD3^+^CD8^+^ cells (right plots) in BALF and lung **(C)** lung immune cell infiltration; and **(D)** serum cytokines/chemokines. Results are presented as individual data points **(A,C)**, pooled data means ± SEM (B), representative images **(C)** and as a heat map showing fold upregulation relative to naive controls **(D)** from two pooled independent experiments (*n* = 8–10) mice per group. Statistical analyses: One-way ANOVA per cell type followed by Dunnett's multiple comparisons test; significant differences relative to unvaccinated mice are indicated by asterisks: *p < 0.05; **p < 0.01; ***p < 0.001; ****p < 0.0001. Original magnification H&E × 25. BCG, Bacille Calmette–Guérin; s.c., subcutaneous; i.t., intratracheal; BALF, bronchioalveolar lavage fluid; LN, lymph node.

No weight loss or other adverse events were observed during the vaccination period in all groups. 60 days after vaccination we analyzed bacterial clearance, immune cell composition, lung pathology, and serum cytokine profiles. In all vaccination settings bacteria were efficiently cleared, and residual BCG colonies, close to the detection limit, were detected in only 5 out of 40 mice (Figure 2A). As previously shown (25), i.t. vaccinations significantly increased the numbers of various T cell subsets in bronchioalveolar lavage fluid (BALF) and lung, including CD44^+^ memory cells and CD69^+^CD103^+^ resident memory T cells (Figure 2B). The largest increase in T cell numbers was observed following i.t. BCGΔBCG1419c vaccination, with a 1.5 to 2.3-fold increase in BALF and a 2.8 to 5.4-fold increase in lungs in comparison to i.t. BCG (CD4^+^CD44^+^ 4.03-fold *p* = 0.0016; CD4^+^CD69^+^CD103^+^ 5.41-fold *p* = 0.0012; CD8^+^CD44^+^ 2.8-fold *p* = 0.0048; CD8^+^CD69^+^CD103^+^ 3.24-fold *p* = 0.0006). Subcutaneous vaccinations induced a modest increase in T cell numbers in spleen, BALF and lung (Supplementary Figure 1A), and no differences in cellularity were detected in the inguinal LN (Supplementary Figure 1A). We also assessed the level of cell infiltration into the lung following the different vaccination regimens by hematoxylin and eosin staining. Compared to naïve mice, the level of cell infiltration after s.c. vaccination was not increased (Figure 2C). Intratracheal vaccinations with both BCG strains led to a low but significant influx of immune cells into the lung which formed organized lymphoid structures, reminiscent of inducible Bronchus Associated Lymphoid Tissue (iBALT) (Figure 2C). Overall, low levels of circulating cytokines and chemokines were detected in all groups, with very few significant differences compared to unvaccinated animals (Figure 2D; Supplementary Figure 1B). Although not reaching statistical significance, relative to s.c. vaccination i.t. vaccination led to reduced upregulation or even a downregulation of many circulating cytokines, including IL-10, IL-6, IL-22, IL-1β, IL-18, and IL-9 (Figure 2D). Collectively, these results demonstrate the safety of all BCG vaccination regimens and indicate a superior capacity of BCGΔBCG1419c to induce memory T cells following i.t. administration.

### BCG Vaccination Prevents Systemic Spread of Reactivated Lymphatic *Mtb* Infection

**Figure 3:**
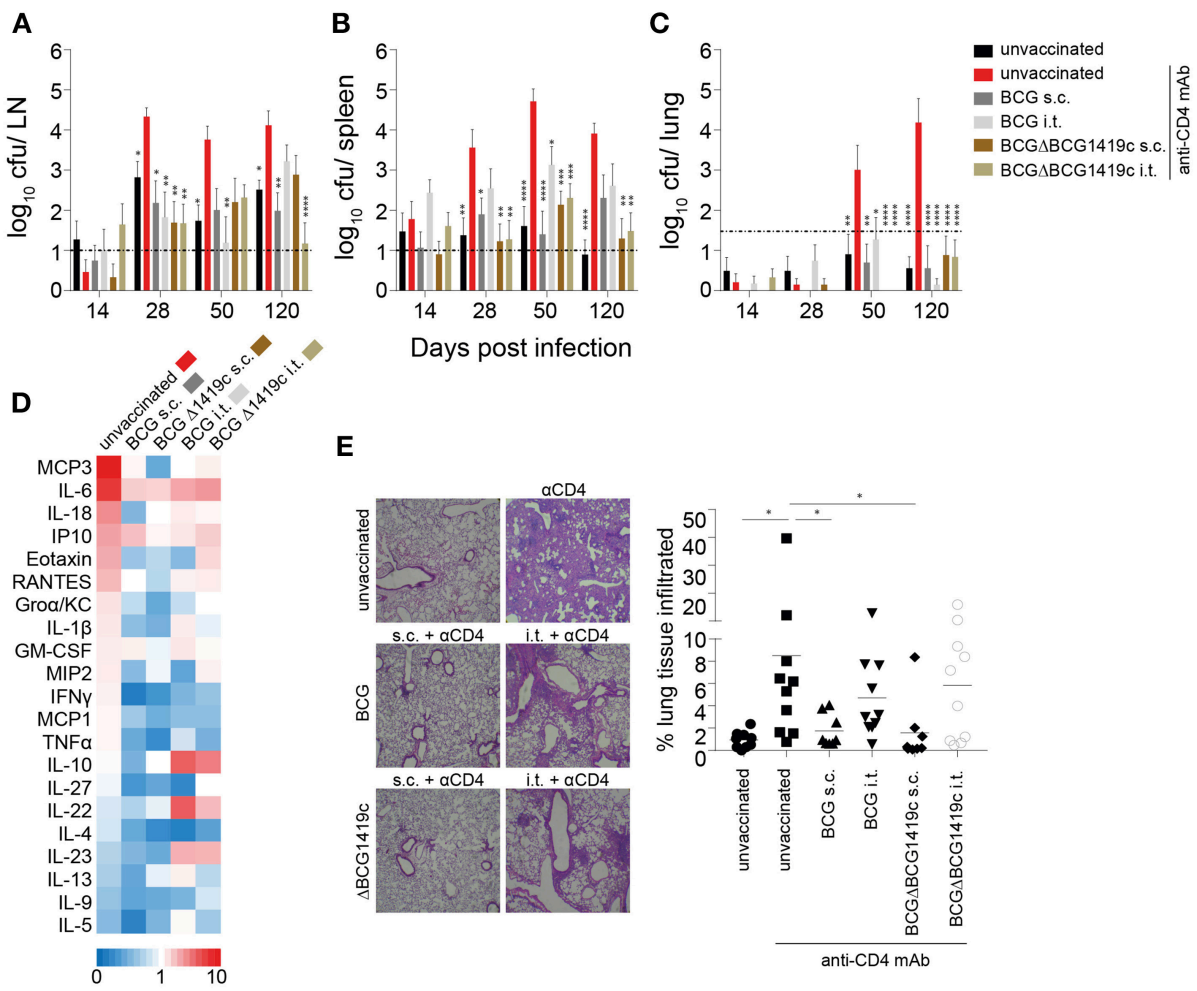
BCG vaccination prevents reactivation of chronic lymphatic *Mtb* infection. Sixty days after vaccination with BCG or BCGΔBCG1419c mice were infected i.d. with 1 × 10^2^ *Mtb* H37Rv. At weekly intervals, mice received an i.p. injection with a mAb against mouse CD4 (GK1.5) or PBS. On days 14, 28, 50, and 120 p.i mice were sacrificed and the ear-draining LNs, spleen and lung were assessed for viable bacteria **(A–C)**. Sera and lung sections from 120 days p.i. were also assessed for cytokines/chemokines **(D)** and immune cell infiltration **(E)**, respectively. Results are presented as pooled data means ± SEM **(A–C)**, individual data points and representative images **(E)** and as a heat map showing fold upregulation relative to unvaccinated untreated controls **(D)** from two pooled independent experiments (*n* = 8–10 mice per group). Statistical analyses: One-way ANOVA per time point followed by Dunnett's multiple comparisons test; significant differences relative to unvaccinated anti CD4-treated mice are indicated by asterisks: *p < 0.05; **p < 0.01; ***p < 0.001; ****p < 0.0001; dotted line in **(A–C)** represents CFU detection limit. Original magnification H&E × 25. BCG, Bacille Calmette–Guérin; s.c., subcutaneous; i.t., intratracheal; LN, lymph node; mAb, monoclonal antibody.

We next assessed if BCG vaccination can prevent the progression from LTBI to active murine TB as a consequence of CD4^+^ T cell depletion. C57BL/6 mice were infected with 100 cfu *Mtb* H37Rv in the ear dermis 60 days after vaccination and thereafter treated weekly with anti-CD4 mAb. Unvaccinated anti-CD4 mAb-treated mice were used as a positive control for reactivation of LTBI. Unvaccinated mice that did not receive anti-CD4 mAb served as a negative control in which *Mtb* remained contained in the LN. We assessed the bacterial burden in ear-draining LNs, spleen and lung at different time points (Figure 1). As previously reported (51), unvaccinated untreated animals almost exclusively contained *Mtb* within the draining LNs of the infected ear with very limited systemic spread. In some animals few bacteria were detected in the spleen but almost never in the lung (Figures 3A–C, black bars). In unvaccinated animals treated with anti-CD4 mAb, *Mtb* not only multiplied significantly within the LNs over time but also exited the ear-draining LNs and spread to spleen and lung in all animals (Figures 3A–C, red bars). Prior vaccination with BCG or BCGΔBCG1419c significantly reduced replication of *Mtb* within the draining lymph nodes and led to a significantly reduced spread of the bacteria to the spleen (Figure 3B, gray and brown bars). Most importantly, all vaccination strategies prevented spread of *Mtb* to the lung following treatment with anti-CD4 mAb (Figure 3C, gray and brown bars). Particularly at 120 days after *Mtb* infection, unvaccinated animals that were treated with anti-CD4 contained an average of 100,000 bacteria in the lung, whereas vaccinated mice consistently showed CFU numbers below or near the detection limit. No consistent patterns were observed, with both BCG strains and vaccination routes being equally protective (Supplementary Table 1). However, particularly during later time points (d120), vaccination with BCGΔBCG1419c led to very low numbers of detectable *Mtb* in spleen and lung, and also in the LN after i.t. vaccination (Figures 3A–C, brown bars).

Consistent with low numbers of detectable bacteria in the lung in BCG vaccinated groups, vaccination also significantly reduced levels of pro-inflammatory cytokines, such as MCP-3, IL-6, IL-18, Eotaxin, and RANTES relative to unvaccinated, anti-CD4 mAb-treated controls (Figure 3D; Supplementary Figure 2). While circulating serum levels of IL-10 and IL-22 were reduced following i.t. vaccination (Figure 2D; Supplementary Figure 1B), these cytokines were upregulated in i.t. vaccinated animals following *Mtb* infection compared to unvaccinated and s.c. vaccinated mice (Figure 3B; Supplementary Figure 2). The levels of immune cell infiltration into the lungs of s.c. vaccinated mice were comparable to unvaccinated untreated mice (Figure 3E). In contrast, unvaccinated mice that had received anti-CD4 mAb showed significant and large-scale lung immunopathology and cell infiltration (Figure 3E). Intratracheally vaccinated animals maintained the level of immune cell infiltration that was observed 60 days after vaccination around the bronchioli, despite showing very low or even undetectable levels of CFU in the lung. The overall infiltration scores of i.t. vaccinated mice were not significantly different to unvaccinated anti-CD4-treated mice. However, in contrast to the diffuse and widespread infiltration seen in anti-CD4-treated unvaccinated animals, the infiltration in i.t. vaccinated animals resembled well-organized and localized iBALT clusters seen prior to *Mtb* infection (Figures 3E, 2C). Together these data demonstrate that BCG vaccination prevents reactivation of latent lymphatic murine TB regardless of the BCG strain used for vaccination. The disparate cytokine levels and immune cell infiltration patterns also suggest that prevention of LTBI reactivation following s.c. and i.t. vaccination may depend on different mechanisms.

### Vaccine-Induced Prevention of Reactivation Is Independent of CD4^+^ T Cells

**Figure 4:**
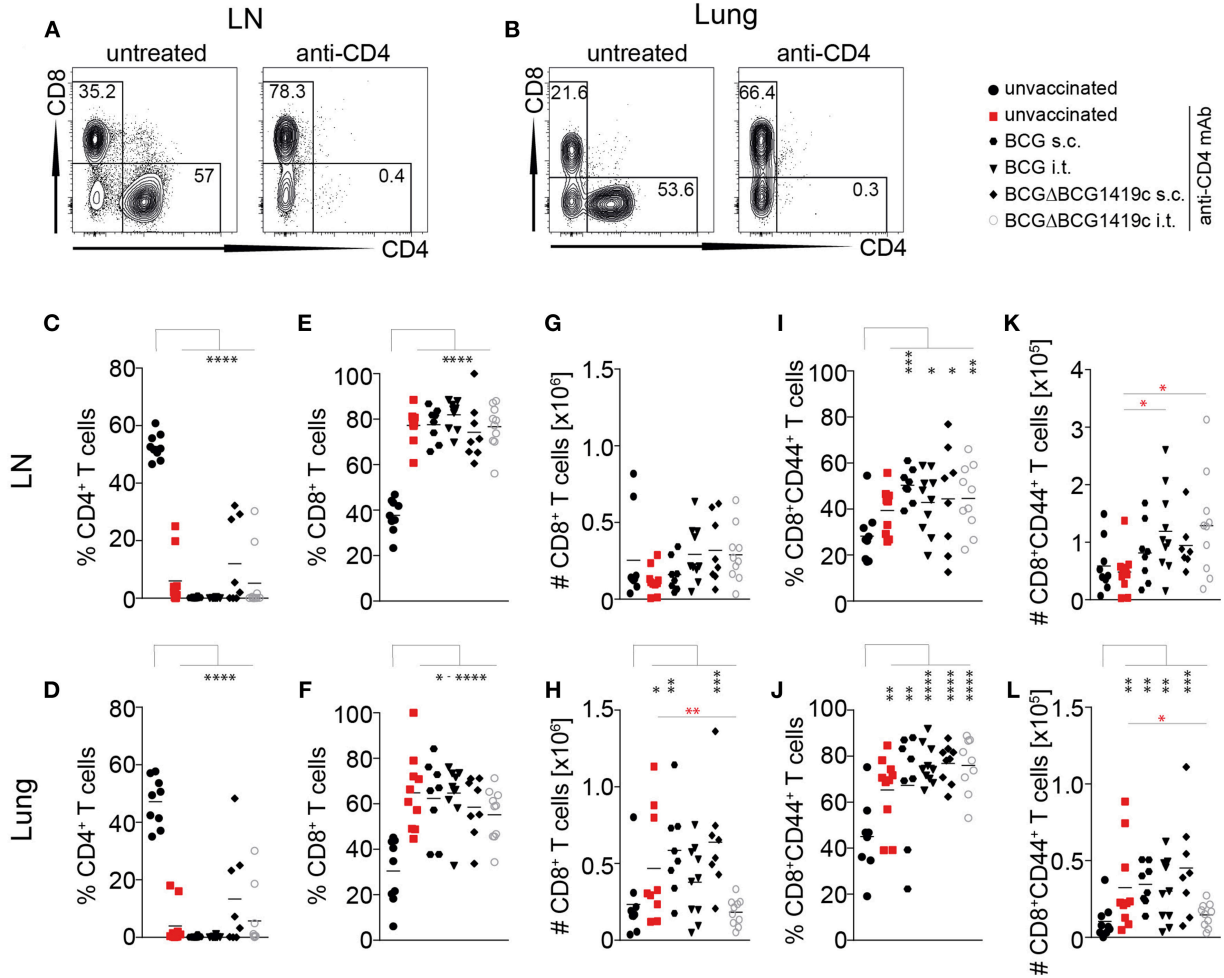
Vaccine-induced prevention of reactivation is independent of CD4^+^ T cells**. (A,B)** Representative FACS plots showing depletion of CD4^+^ T cells in LN and Lung (gated on CD3^+^ cells) following administration of anti-CD4 mAb. **(C,D)** Frequencies of CD3^+^CD4^+^ cells in LN **(C)** and lung **(D)** at 120 days following *Mtb* infection. **(E,F)** Frequencies of CD3^+^CD8^+^ cells in LN **(E)** and lung **(F)** at 120 days following *Mtb* infection. **(G,H)** Total numbers of CD3^+^CD8^+^ cells in LN and lung at 120 days following *Mtb* infection. **(I,J)** Frequencies of CD3^+^CD8^+^CD44^+^ cells in LN and lung at 120 days following *Mtb* infection. **(K,J)** Total number of CD3^+^CD8^+^CD44^+^ cells in LN and lung at 120 days following *Mtb* infection. Results are presented as representative FACS plots **(A,B)** or individual data points **(C–L)** from two pooled independent experiments (*n* = 8–10 mice per group). Statistical analyses: one-way ANOVA followed by Dunnett's multiple comparisons test; significant differences relative to unvaccinated mice are indicated by black asterisks; statistical differences relative to unvaccinated anti-CD4-treated mice are indicated by red asterisks: *p < 0.05; **p < 0.01; ***p < 0.001; ****p < 0.0001. BCG, Bacille Calmette–Guérin; s.c., subcutaneous; i.t., intratracheal; LN, lymph node; mAb, monoclonal antibody.

Given that anti-CD4 treatment in unvaccinated mice (Figures 3A–C, red bars) led to reactivation of LTBI, we also investigated the efficiency of CD4^+^ T cell depletion. In all groups, anti-CD4 mAb treatment led to almost complete depletion of CD4^+^ T cells from ear-draining lymph nodes as well as from the lung parenchyma (Figures 4A–D). Depletion efficacy progressively increased from day 14 (Supplementary Figures 3A,B), and by day 120 after infection only 7 out of 50 mice showed low residual levels of CD4^+^ T cells in lymph nodes and lung (Figures 4C,D). In line with effective depletion of CD4^+^ T cells, the frequency of CD8^+^ T cells significantly increased in LN and lung of all anti-CD4-treated mice (Figures 4E,F; Supplementary Figures 3C,D). However, this proportional increase was initially not accompanied by a numerical increase, because at days 14, 28 and 50 p.i. total CD8^+^ T cell numbers in LN and lung were not statistically significant between groups (Supplementary Figures 3E,F). Only the anti CD4-treated unvaccinated group, the s.c. BCG group and the s.c. BCGΔBCG1419c group reached statistical significance in the lung but not the LN at day 120 p.i. (Figures 4G,H). Similarly, while CD44^+^ memory CD8^+^ T cells proportionally increased following anti-CD4 mAb treatment (Figures 4I,J; Supplementary Figures 3G,H), statistically significant numerical increases were only observed at day 120 p.i. in the lung (Figures 4K,L; Supplementary Figures 3I,J). These results suggest that depletion of CD4^+^ T cells does not lead to a numerical replacement by CD8^+^ T cells to control infection in the LN and lung. An additional statistical comparison between unvaccinated anti-CD4-treated mice with anti-CD4-treated vaccinated groups (red asterisks in Figure 4 and Supplementary Figure 3), revealed that the proportional increase in CD8^+^ T cells in the lung of vaccinated mice was lower in almost all vaccination groups at days 14, 28, and 50 after *Mtb* infection (Supplementary Figure 3D). These differences were not observed at 120 days after challenge and did not translate into statistically significant numerical differences in total CD8^+^ T cell numbers (Supplementary Figure 3F) or memory CD8^+^ T cell numbers (Supplementary Figure 3J) for most vaccination groups. Compared to unvaccinated anti-CD4-treated mice, only i.t. BCGΔBCG1419c vaccinated mice showed significantly reduced numbers in total CD8^+^ (Figure 4H) and memory CD8^+^ T cells numbers (Figure 4L) in the lung 120 days after *Mtb* challenge. Furthermore, when we assessed memory CD8^+^ T cells for the expression of CD62L, CD69, and CD103 to compare frequencies of effector-, central-, and resident memory T cells, no obvious differences between the vaccination groups and unvaccinated control groups were observed. However, the increase in resident memory T cells that was only induced by i.t. vaccination (Figure 2B) was maintained following *Mtb* challenge in the lung (Supplementary Figure 4A). Overall these data unambiguously demonstrate that BCG vaccination prevents LTBI reactivation without a requirement for CD4^+^ T cells, and suggest that numerical changes in CD8^+^ T cells are unlikely to mediate the prevention of LTBI reactivation in this model.

## Discussion

Using a tractable mouse model, we provide compelling evidence that immune control of reactivation of latent lymphatic *Mtb* infection can be independent of vaccine-induced CD4^+^ T cells. Treatment of latently infected mice with anti-CD4 mAb caused LTBI reactivation and systemic spread of *Mtb* to spleen and lung, mimicking the rapid progression from LTBI to TB in HIV^+^ individuals (51). In contrast, prior vaccination with BCG or BCGΔBCG1419c prevented reactivation of *Mtb* from the ear-draining lymph nodes and systemic spread, regardless of the route of vaccine delivery. As most mice were depleted of CD4^+^ T cells, our results unambiguously demonstrate that vaccine-induced non-CD4^+^ T cell responses are sufficient to control latent lymphatic *Mtb* infection. Furthermore, we provide evidence that the experimental TB vaccine BCGΔBCG1419c is safe and induces more memory T cells in BALF and lung tissue following i.t. vaccination. Collectively, these results further highlight the incomplete understanding about what constitutes immunity in TB and why BCG only efficiently prevents some forms of TB but not others (7, 8).

As expected, the frequency of CD8^+^ T cell in the infected lymph nodes and lungs increased following the depletion of CD4^+^ T cells. However, the total number of CD8^+^ T cells only significantly increased in the lungs of unvaccinated anti-CD4-treated mice and s.c. vaccinated mice at 120 days after *Mtb* infection. Similarly, while the frequency of CD44^+^ memory CD8^+^ T cells increased after depletion of CD4^+^ T cells, the total numbers of these cells were only significantly increased at 120 days after *Mtb* infection in the lung of unvaccinated anti-CD4-treated mice, s.c. vaccinated mice and i.t. BCG vaccinated mice. Importantly, i.t. vaccination with BCGΔBCG1419c did not significantly increase CD8^+^ T cell populations, but even significantly reduced CD8^+^ T cell populations relative to unvaccinated anti-CD4-treated mice, despite providing robust long-term protection against systemic spread of *Mtb*. These results suggest that protection from reactivation is unlikely to be exclusively mediated by an increase in CD8^+^ T cell populations, but may perhaps rely on BCGΔBCG1419c's improved capacity to stimulate local CD8^+^ IFNγ^+^ T lymphocytes in response to *Mtb* antigens, as previously reported (29). This is consistent with our previous study in which we had investigated if the boost of memory CD8^+^ T cells, DN T cells, and NK cells with IL-2/anti-IL-2 complexes can reverse the reactivation of lymphatic LTBI. Although IL-2/anti-IL-2 complex treatment significantly expanded these immune cell populations in LN, spleen and lung, it did not prevent the systemic spread of *Mtb* after CD4^+^ T cells were depleted (51). It was also recently shown that CD8^+^ T cells fail to recognize *Mtb*-infected macrophages, due to bacterial-induced decoy mechanism, using distinct immunodominant *Mtb* antigens in an *in vitro* model (61). Further studies are needed to investigate the role of antigen-specific CD8^+^ T cells and their function in localized IFN-γ production in this model. On the other hand, it could be that such protection from reactivation of lymphatic LTBI could be mediated by activated macrophages, which were shown to be more abundant in another model of reactivation from latent-like infection upon corticosteroid treatment, as opposed to IFNγ^+^ T cells (29). An increased turnover rate of tissue macrophage was also suggested to be important in TB reactivation in rhesus macaques (12). Further studies will be required to formally confirm or to rule out these hypotheses.

It was beyond the scope of this study to investigate which immune mechanisms contain *Mtb* infection in the absence of CD4^+^ T cells. Hence, we did not further investigate humoral immunity, B cell characteristics or innate (trained) immune responses. The importance of such immune responses in TB has recently received renewed attention (62, 63). In particular the distinct antibody glycosylation pattern identified in human LTBI (64) has highlighted a potentially important role for antibody-mediated effector function in controlling latent infection. Furthermore, there is increasing evidence that *Mtb* exposure generates antibody isotypes in humans that can inhibit mycobacterial infection (65), particularly in previously uninfected individuals and individuals with LTBI (66). It is also interesting to note that patients with active TB appear to have dysfunctional circulating B cells, which regain function following successful treatment (67). In support of our study, it was also recently demonstrated that reactivation of LTBI in a macaque model of TB/SIV co-infection was independent of CD4^+^ T cells in at least one third of all animals (11). Collectively these results point towards an important role of B cell-mediated immunity in LTBI control as well as prevention of *Mtb* infection. Our study extends these findings, and it is hence tempting to speculate that BCG vaccination may prevent reactivation of LTBI via B cell-mediated or trained innate immunity. The tractable, reproducible and widely affordable mouse model presented here, will allow to study the contributions of these mechanisms on the dynamics of LTBI reactivation in future studies.

The recombinant BCGΔBCG1419c has previously been shown to protect against *Mtb* challenge in chronic TB models (29, 37). Our study also provides evidence that BCGΔBCG1419c induces equivalent protection in this model of LTBI reactivation compared to conventional BCG against dissemination to the lungs, and improved reduction of *Mtb* replication in LN and spleens at the latest time point evaluated in this work, 120 days post-infection (Figures 3A,C). Intratracheal administration of BCGΔBCG1419c induced significantly higher numbers of memory T cells in the lung, including cells with resident memory phenotype. Mucosal vaccination with BCGΔBCG1419c also induces significantly higher levels of organized lymphoid structures in the lung, similar to iBALT. Although these findings do not explain why subcutaneous vaccination also prevents systemic spread of *Mtb* in this model, there is increasing evidence that lymph node architecture also changes significantly toward a B cell-dominated structure following infection (68). Taken together, it is possible that both s.c. and i.t. BCG vaccinations protect against reactivation of LTBI by anatomically distinct but functionally similar mechanisms that are geared towards expansion of B cells. Further studies will have to investigate these hypotheses.

Future investigations should also focus on determining whether a LTBI model in genetically modified mouse strains lacking particular immune cell subsets can be established. A combination of different KO mouse strains and different vaccines may ultimately reveal which cell type contains *Mtb* in the absence of CD4^+^ T cells. These studies should be accompanied by detailed, high-dimensional imaging analyses of the immune cell compositions in infected lymph nodes, spleen and lung to dissect whether containment of *Mtb* largely occurs in the infected lymph node or via preferential killing by vaccine-induced immune cell subsets in the spleen and/or lung. The time-dependent spread of *Mtb* from LN to spleen and lung following reactivation with anti-CD4 suggests the presence of highly coordinated tissue-specific immune responses that may involve macrophages and other antigen presenting cells. Additionally, the absence of *Mtb* CFU in the lung of vaccinated animals may also suggest that the spread of *Mtb* from the lymphatics to the lung is interrupted, and that vaccine-induced containment predominantly occurs in the infected lymph nodes and the spleen. All of these hypotheses will require further investigation.

In summary, our results provide compelling evidence that BCG vaccination protects against reactivation of LTBI independently of vaccine-induced CD4^+^ T cells. Our findings suggest that protection from reactivation may be independent of CD8^+^ T cell expansion and suggest a potential role for B cells, antibody and/or trained immunity in preventing reactivation. Finally, these results also underpin the importance of our new small animal model of LTBI to gain new insights into the correlates of BCG-induced immunity against *Mtb*.

## Ethics Statement

All experiments were approved and conducted according to Australian animal protection law and in accordance with requirements by the animal ethics committee of James Cook University (A2403).

## Author Contributions

AK and MF-V conceived of the study. MF-V designed and generated the BCGΔBCG1419c strain. AK, HS, MA-S, and SP performed experiments. AK and HS performed data analysis and wrote the manuscript. NK and MF-V commented extensively on the manuscript. All coauthors read the manuscript and approved it.

### Conflict of Interest Statement

The authors declare that the research was conducted in the absence of any commercial or financial relationships that could be construed as a potential conflict of interest.
