# Supplementary material for: BCG Vaccination Prevents Reactivation of Latent Lymphatic Murine Tuberculosis Independently of CD4+ T Cells
Source: Front Immunol. 2019 Mar 21;10:532. doi: 10.3389/fimmu.2019.00532 (PMC6437071; doi:10.3389/fimmu.2019.00532)
Supplement: Supplementary file 1 [file Data_Sheet_1.pdf]

frontiers

Supplementary Material

BCG vaccination prevents reactivation of latent lymphatic murine tuberculosis independently of $CD4^+$ T cells

Harindra D. Sathkumara[1], Saparna Pai[1], Michel de Jesús Aceves-Sánchez[2], Natkunam Ketheesan[3], Mario Alberto Flores-Valdez[2, #] & Andreas Kupz[1, #]

*** Correspondence:**
Andreas Kupz
andreas.kupz@jcu.edu.au

Mario Alberto Flores Valdez
floresv@ciatej.mx

Supplementary Figures and Tables

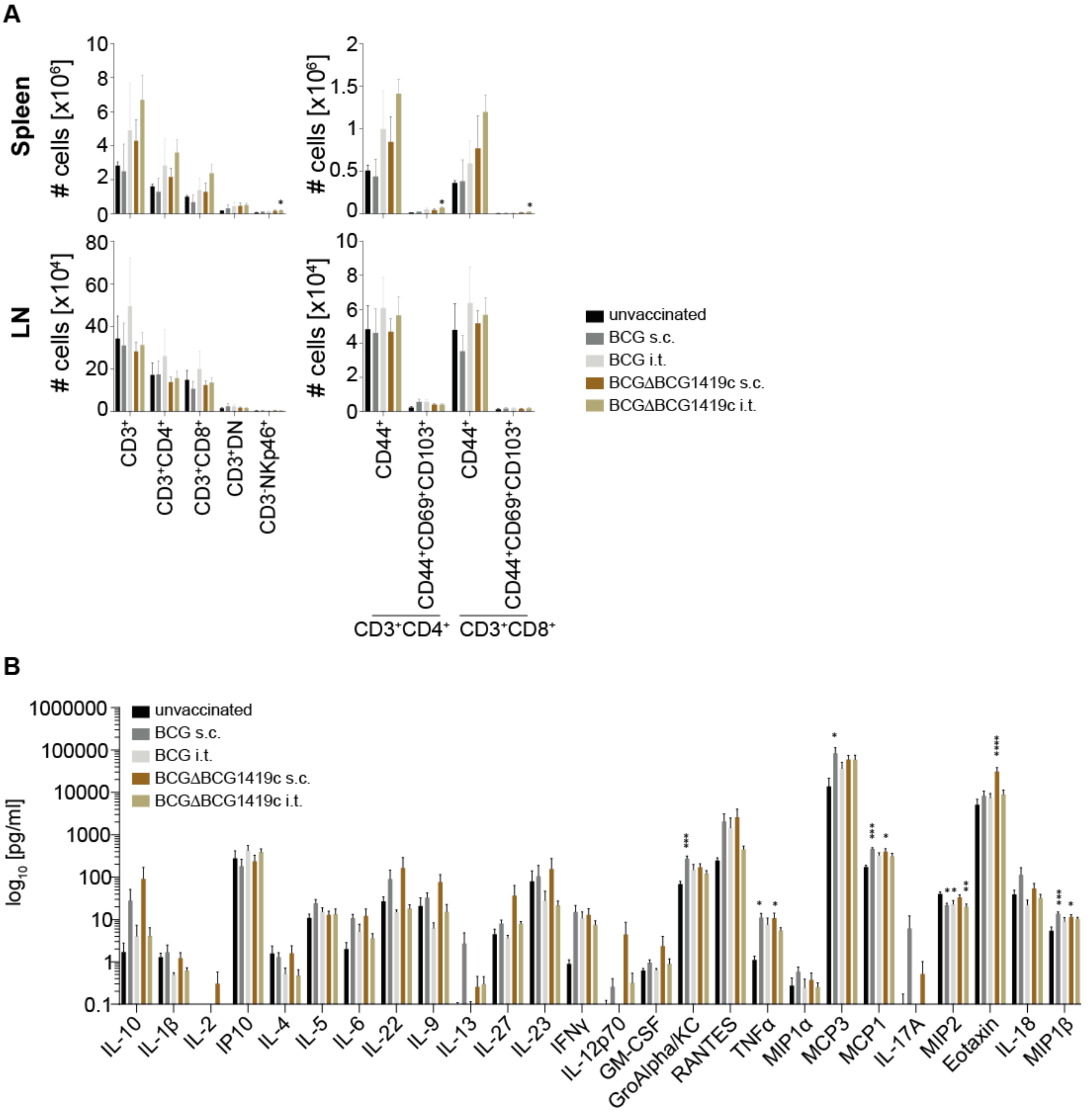

Supplementary Figure 1: T cell numbers, serum cytokine and chemokine levels prior to *Mtb* infection. (A) Number of total CD3$^+$, CD3$^+$CD4$^+$, CD3$^+$CD8$^+$, CD3$^+$CD4$^-$CD8$^-$ (DN) and CD3$^-$NKp46$^+$ cells (left plots), as well as numbers of total CD44$^+$, CD44$^+$CD69$^+$CD103$^+$ cells amongst CD3$^+$CD4$^+$ and CD3$^+$CD8$^+$ cells (right plots) in spleen and inguinal LN 60 days after vaccination with BCG or BCGΔBCG1419c. (B) Levels of serum cytokines and chemokines at 60 days after vaccination. Results are presented as pooled data means ± SEM from two pooled independent experiments (n = 8–10 mice per group). Statistical analyses: One-way ANOVA per analyte followed by Dunnett's multiple comparisons test; significant differences relative to unvaccinated mice are indicated by asterisks: * p<0.05; ** p<0.01; *** p<0.001; **** p<0.0001. Abbreviations: BCG, Bacille Calmette–Guérin; s.c., subcutaneous; i.t., intratracheal; (Supplementary to Fig. 2)

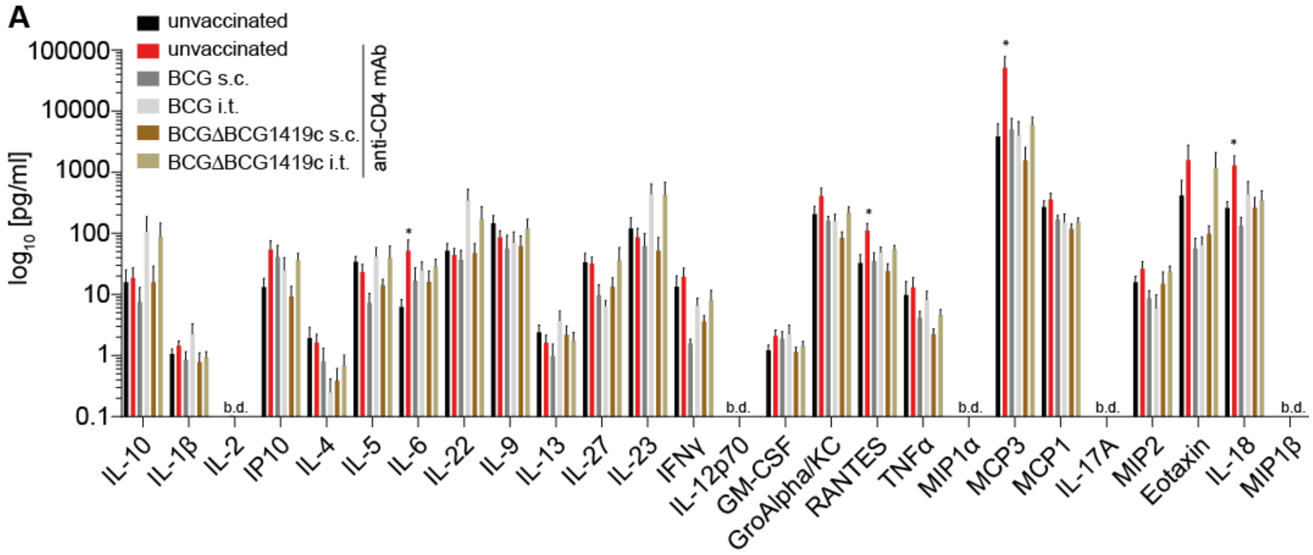

Supplementary Figure 2: Serum cytokine and chemokine levels 120 days after *Mtb* infection. (A) Levels of serum cytokines and chemokines at 120 days after *Mtb* infection. Results are presented as pooled data means ± SEM from two pooled independent experiments (n = 8–10 mice per group). Statistical analyses: One-way ANOVA per analyte followed by Dunnett's multiple comparisons test; significant differences relative to unvaccinated mice are indicated by asterisks: * $p<0.05$; b.d. below detection limit. Abbreviations: BCG, Bacille Calmette–Guérin; s.c., subcutaneous; i.t., intratracheal; mAb, monoclonal antibody (Supplementary to Fig. 3)

Supplementary Figure 3

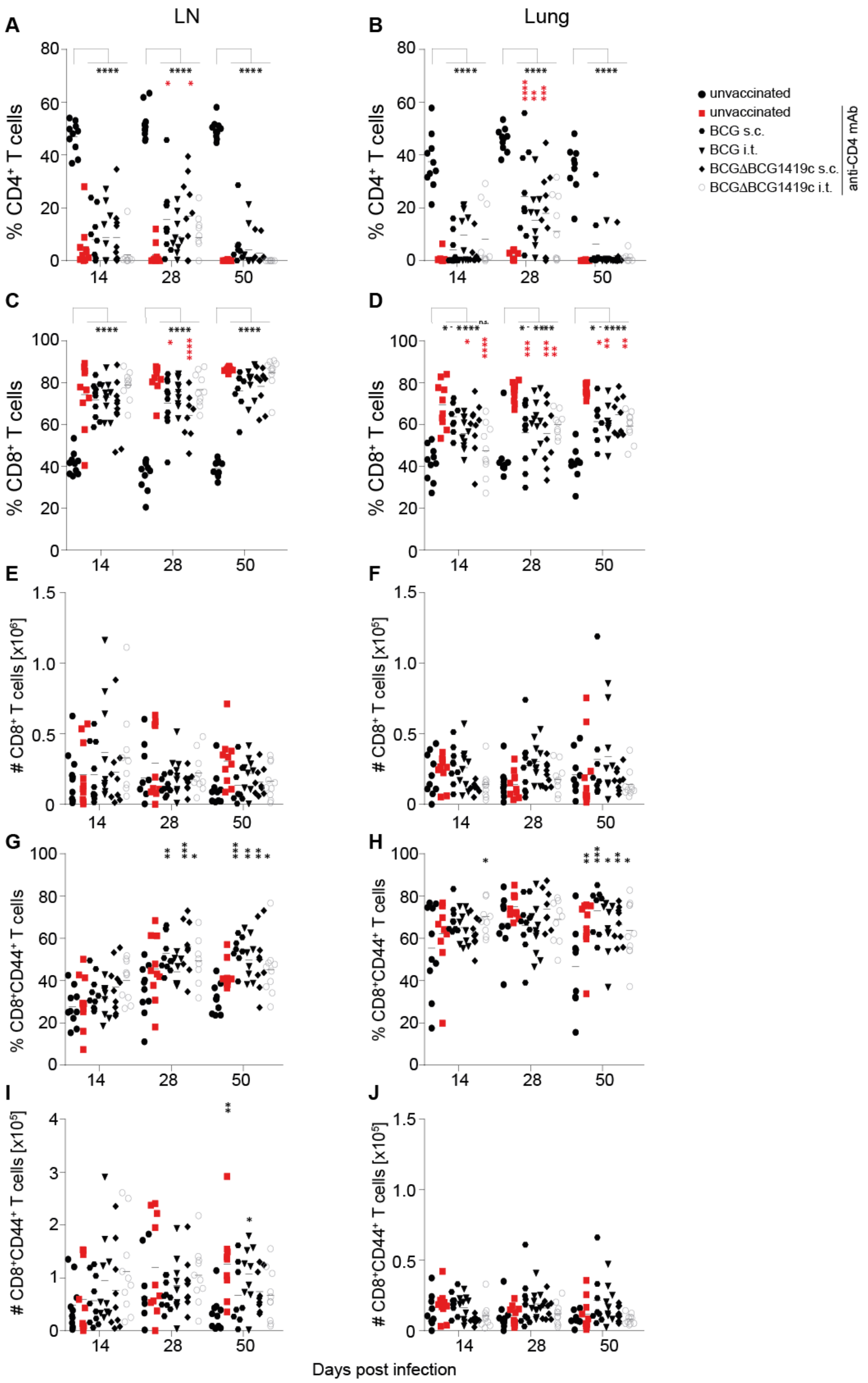

Supplementary Figure 3: CD8+ T cell profiling after *Mtb* infection. (A, B) Frequencies of CD3+CD4+ cells in LN (A) and lung (B) at 14, 28 and 50 days following *Mtb* infection. (C, D) Frequencies of CD3+CD8+ cells in LN (C) and lung (D) at 14, 28 and 50 days following *Mtb* infection. (E, F) Total numbers of CD3+CD8+ cells in LN and lung at 14, 28 and 50 days following *Mtb* infection. (G, H) Frequencies of CD3+CD8+CD44+ cells in LN and lung at 14, 28 and 50 days following *Mtb* infection. (I, J) Total number of CD3+CD8+CD44+ cells in LN and lung at 14, 28 and 50 days following *Mtb* infection. Results are presented as individual data points from two pooled independent experiments (n = 8–10 mice per group). Statistical analyses: One-way ANOVA per time point followed by Dunnett's multiple comparisons test; significant differences relative to unvaccinated mice are indicated by black asterisks; statistical differences relative to unvaccinated anti-CD4-treated mice are indicated by red asterisks: * $p<0.05$; ** $p<0.01$; *** $p<0.001$; **** $p<0.0001$. Abbreviations: BCG, Bacille Calmette–Guérin; s.c., subcutaneous; i.t., intratracheal; LN, lymph node; mAb, monoclonal antibody (Supplementary to Fig. 4)

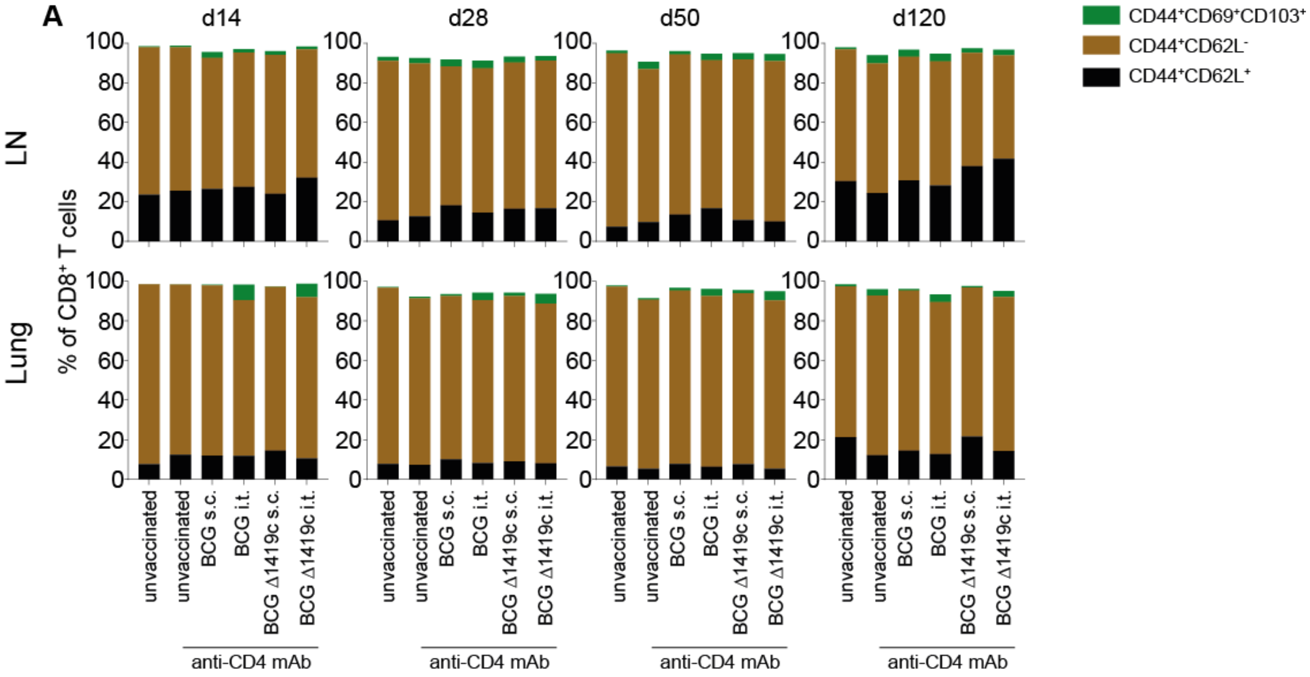

Supplementary Figure 4: Memory CD8+ T cell profiling after *Mtb* infection. (A) Frequencies of CD44+CD62L-, CD44+CD62L+ and CD44+CD69+CD103+ cells amongst CD8+ T cells. Results are presented as pooled data means from two pooled independent experiments (n = 8–10 mice per group). Statistical analyses: Abbreviations: BCG, Bacille Calmette–Guérin; s.c., subcutaneous; i.t., intratracheal; LN, lymph node; mAb, monoclonal antibody (Supplementary to Fig. 4)

LN d14	unvacc.	anti CD4	BCG s.c.	BCG i.t.	Δ1419c s.c.	Δ1419c i.t.
unvacc.		0.758	0.9516	0.998	0.6265	0.9892
anti CD4			0.9969	0.9409	>0.9999	0.3756
BCG s.c.				0.9978	0.9823	0.6718
BCG i.t.					0.8663	0.8988
Δ1419c s.c.						0.2637
Δ1419c i.t.						

SPL d14	unvacc.	anti CD4	BCG s.c.	BCG i.t.	Δ1419c s.c.	Δ1419c i.t.
unvacc.		0.9928	0.9747	0.4905	0.8973	0.9999
anti CD4			0.7759	0.8309	0.5897	0.9996
BCG s.c.				0.1356	0.9996	0.9162
BCG i.t.					0.0682	0.6508
Δ1419c s.c.						0.7816
Δ1419c i.t.						

Lung d14	unvacc.	anti CD4	BCG s.c.	BCG i.t.	Δ1419c s.c.	Δ1419c i.t.
unvacc.		0.9095	0.4945	0.8678	0.4945	0.9909
anti CD4			0.9747	>0.9999	0.9747	0.9982
BCG s.c.				0.9873	>0.9999	0.8478
BCG i.t.					0.9873	0.9946
Δ1419c s.c.						0.8478
Δ1419c i.t.						

LN d28	unvacc.	anti CD4	BCG s.c.	BCG i.t.	Δ1419c s.c.	Δ1419c i.t.
unvacc.		0.498	0.9355	0.6945	0.568	0.5537
anti CD4			0.0117	0.0026	0.0014	0.0013
BCG s.c.				0.9952	0.979	0.976
BCG i.t.					>0.9999	>0.9999
Δ1419c s.c.						>0.9999
Δ1419c i.t.						

SPL d28	unvacc.	anti CD4	BCG s.c.	BCG i.t.	Δ1419c s.c.	Δ1419c i.t.
unvacc.		0.0049	0.9614	0.4416	>0.9999	>0.9999
anti CD4			0.0457	0.3687	0.0025	0.0031
BCG s.c.				0.9067	0.8959	0.9219
BCG i.t.					0.3107	0.3511
Δ1419c s.c.						>0.9999
Δ1419c i.t.						

Lung d28	unvacc.	anti CD4	BCG s.c.	BCG i.t.	Δ1419c s.c.	Δ1419c i.t.
unvacc.		0.9038	0.6787	0.9749	0.9038	0.6787
anti CD4			0.9977	0.4853	>0.9999	0.9977
BCG s.c.				0.2429	0.9977	>0.9999
BCG i.t.					0.4853	0.2429
Δ1419c s.c.						0.9977
Δ1419c i.t.						

LN d50	unvacc.	anti CD4	BCG s.c.	BCG i.t.	Δ1419c s.c.	Δ1419c i.t.
unvacc.		0.0128	0.9986	0.9632	0.9812	0.945
anti CD4			0.0559	0.0015	0.0894	0.115
BCG s.c.				0.8532	0.9998	0.9976
BCG i.t.					0.68	0.544
Δ1419c s.c.						>0.9999
Δ1419c i.t.						

SPL d50	unvacc.	anti CD4	BCG s.c.	BCG i.t.	Δ1419c s.c.	Δ1419c i.t.
unvacc.		0.0001	0.9993	0.1203	0.945	0.8257
anti CD4			0.0001	0.0417	0.0003	0.0006
BCG s.c.				0.0781	0.8427	0.6718
BCG i.t.					0.5751	0.728
Δ1419c s.c.						0.9997
Δ1419c i.t.						

Lung d50	unvacc.	anti CD4	BCG s.c.	BCG i.t.	Δ1419c s.c.	Δ1419c i.t.
unvacc.		0.0042	0.9995	0.9906	0.6839	0.6588
anti CD4			0.003	0.0283	0.0001	0.0001
BCG s.c.				0.9495	0.8879	0.8774
BCG i.t.					0.3468	0.3189
Δ1419c s.c.						>0.9999
Δ1419c i.t.						

LN d120	unvacc.	anti CD4	BCG s.c.	BCG i.t.	Δ1419c s.c.	Δ1419c i.t.
unvacc.		0.0351	0.9548	0.8294	0.9902	0.2145
anti CD4			0.0041	0.3843	0.1696	0.0001
BCG s.c.				0.3297	0.7149	0.7568
BCG i.t.					0.9935	0.009
Δ1419c s.c.						0.0669
Δ1419c i.t.						

SPL d120	unvacc.	anti CD4	BCG s.c.	BCG i.t.	Δ1419c s.c.	Δ1419c i.t.
unvacc.		0.0001	0.3001	0.0902	0.9905	0.9373
anti CD4			0.0695	0.1481	0.001	0.0012
BCG s.c.				0.9969	0.6876	0.8044
BCG i.t.					0.3488	0.4524
Δ1419c s.c.						0.9997
Δ1419c i.t.						

Lung d120	unvacc.	anti CD4	BCG s.c.	BCG i.t.	Δ1419c s.c.	Δ1419c i.t.
unvacc.		0.0001	>0.9999	0.9839	0.9957	0.9974
anti CD4			0.0001	0.0001	0.0001	0.0001
BCG s.c.				0.986	0.9978	0.8479
BCG i.t.					0.8479	0.8529
Δ1419c s.c.						>0.9999
Δ1419c i.t.						

Supplementary Table 1: P-values for CFU results presented in Figure 3A-C. A one-way ANOVA per time point followed by Tukey’s multiple comparisons test was performed for CFU results shown in Figure 3A. The mean of each group was compared to the mean of every other group and p values for each combination and time point are shown. (Supplementary to Figure 3)
